# Effect of m-health-based core stability exercise combined with self-compassion training for patients with non-specific chronic low back pain: study protocol for a randomized controlled trial

**DOI:** 10.1186/s13063-022-06258-0

**Published:** 2022-04-07

**Authors:** Zheng Fuming, Xiao Weihui, Yang Jiajia, Liu Shufeng, Zheng Yiyi, Liang Wenjian, Li Yan, Li Zhicheng, Zhang Siyun, Zou Yingmin, Wang Yuyin, Wang Chuhuai

**Affiliations:** 1 Department of Rehabilitation Medicine, The First Affiliated Hospital, Sun Yat-sen University, Guangzhou, 510080 China; 2 Department of Psychology, Sun Yat-sen University, Guangzhou, 510006 China

**Keywords:** Non-specific chronic low back pain, Core stability exercise, Self-compassion training, Mobile health

## Abstract

**Background:**

Non-specific chronic low back pain (NCLBP) has a high incidence, which has a significant impact on a patient’s body and mind and is a common condition affecting people’s quality of life. Core stability exercise (CSE) is a modestly effective treatment for NCLBP; however, CSE has only been shown to be a useful treatment option in the short term. Many clinical practice guidelines recommend the use of a biopsychosocial framework to guide the management of NCLBP. Self-compassion training (SCT) is a promising psychotherapy treatment option for NCLBP; however, there is still a lack of research on CSE combined with SCT. In this study, we will seek to determine whether CSE combined with SCT is an effective treatment option for patients with NCLBP compared to CSE alone.

**Methods:**

In this study, we will randomize 166 adults with NCLBP to a combined SCT and CSE arm or a CSE alone arm (83 participants per group). Both interventions will consist of four weekly 1.5-h group sessions of CSE supplemented by home practice. The combined group protocol also includes 2 h of SCT before CSE. Interviewers masked to the treatment assignments will assess the outcomes at 4 and 16 weeks post-randomization. The primary outcomes are back pain disability (based on the Roland-Morris Disability Questionnaire) and pain intensity (NRS; average pain, worst pain, average pain) at 16 weeks.

**Discussion:**

If SCT is found to enhance the effectiveness of CSE for patients with chronic back pain, the results of the study may promote the development of mind-body therapies for chronic low back pain.

**Trial registration:**

Chinese Clinical Trial Registry ChiCTR2100042810. Registered on 21 January 2021

## Administrative information

Note: The numbers in curly brackets in this protocol refer to SPIRIT checklist item numbers. The order of the items has been modified to group similar items (see http://www.equator-network.org/reporting-guidelines/spirit-2013-statement-defining-standard-protocol-items-for-clinical-trials/).

**Table:** 

Title {1}	Effect of m-health-based core stability exercise combined with self-compassion training for patients with non-specific chronic low back pain: study protocol for a randomized controlled trial
Trial registration {2a and 2b}.	The trial was prospectively registered with the Chinese Clinical Trial Registry Number: ChiCTR2100042810. Registered on 21 Jan 2021.
Protocol version {3}	Issue Date: 30 August 2021 Protocol Amendment Number: 05
Funding {4}	The article processing charge will be funded by the National Natural Science Foundation of China (82172532). The correlative charges of fMRI will be funded by the National Natural Science Foundation of China (82172532) and the Young Scientists Fund (82002398).
Author details {5a}	ZFM conceived the study. XWH, ZYM, WYY, WCH, YJJ, LSF, and LZC initiated the study design. LWJ and LY helped with the implementation. WCH and ZSY are grant holders. WYY provided statistical expertise in the clinical trial design. LZC is conducting the primary statistical analysis. All authors contributed to the refinement of the study protocol and approved the final manuscript. WYY and WCH held full responsibility for this manuscript equally.
Name and contact information for the trial sponsor {5b}	Wang Chuhuai (wangchuh@mail.sysu.edu.cn) Dr Zhang Siyun (zhangsy63@mail.sysu.edu.cn)
Role of sponsor {5c}	The funding sources had no role in the design of this study and will not have any role during its execution, analyses, interpretation of the data, or decision to submit results.

## Introduction

### Background and rationale {6a}

Low back pain is a very common condition that occurs in people in different regions (developed and developing countries) and at different ages (from children to the elderly) [1]. According to the 2019 Global Burden of Disease Study, low back pain was identified as one of the main conditions affecting the health level of young and middle-aged people [2]. The most common form of low back pain is non-specific low back pain. This term is used when the pathoanatomical cause of the pain cannot be determined [3]. Non-specific chronic low back pain (NCLBP), defined as pain lasting for 12 weeks or longer [4], can cause not only physical suffering, but also psychological and social problems [5]. Although there is a vast range of physical and psychological therapies to treat NCLBP, the social burden associated with NCLBP has been increasing [6].

The lumbar multifidus (LM) and transversus abdominis (TrA) are deep stabilizing spinal muscles that play crucial roles in the lumbar spine [7]. Studies have reported that there is significant atrophy, fat infiltration, and/or decrease in activation of the TrA and LM muscles in individuals with NCLBP [8, 9]. Core stability exercises (CSEs) can increase the activation of LM and TrA and have been proven useful for treating NCLBP [10]. However, patients with NCLBP exhibit poor adherence to exercise training without supervision, leading to poor efficiency [11, 12]. In addition to the core muscles, low back pain may be related to psychological factors, such as perceptions of back pain, depression, fear of activity, and pain self-efficacy [13]. Additionally, there are many proven effective psychotherapy methods for treating NCLBP, such as mindfulness-based stress reduction (MBSR). Relevant trials have suggested that MBSR may be an effective treatment option for patients with chronic low back pain [14–16]. Self-compassion has been proposed as a key mediator of mindfulness-based interventions [17]. An exploratory pilot study suggested that brief self-compassion training (SCT) could help people with NCLBP by reducing clinical pain intensity and disability and could increase trait self-compassion and interoceptive awareness [18]. Importantly, SCT can increase the practice of health-promoting behaviors [19]. Therefore, the addition of SCT to CSE may improve the treatment effect of low back pain. However, there is still a lack of relevant evidence about the integration of SCT and CSE to treat NCLBP. It needs to be validated by the implementation of high-quality randomized controlled trials.

### Objectives {7}

Our specific aims and their corresponding hypotheses are outlined below as follows:

1. To evaluate the effectiveness of SCT combined with CSE compared to CSE alone for NCLBP

*Hypothesis 1:* SCT can reduce the negative emotions of patients with NCLBP and increase their adherence with CSE, thus achieving relief of pain intensity and disability.

2. To explore the changes in brain function and muscle structure after treatment

*Hypothesis 2:* Activation of the insula will be observed after SCT. Activation of the core stabilizing muscles (LM and TA) will be observed after CSE.

### Trial design {8}

**Fig. 1:**
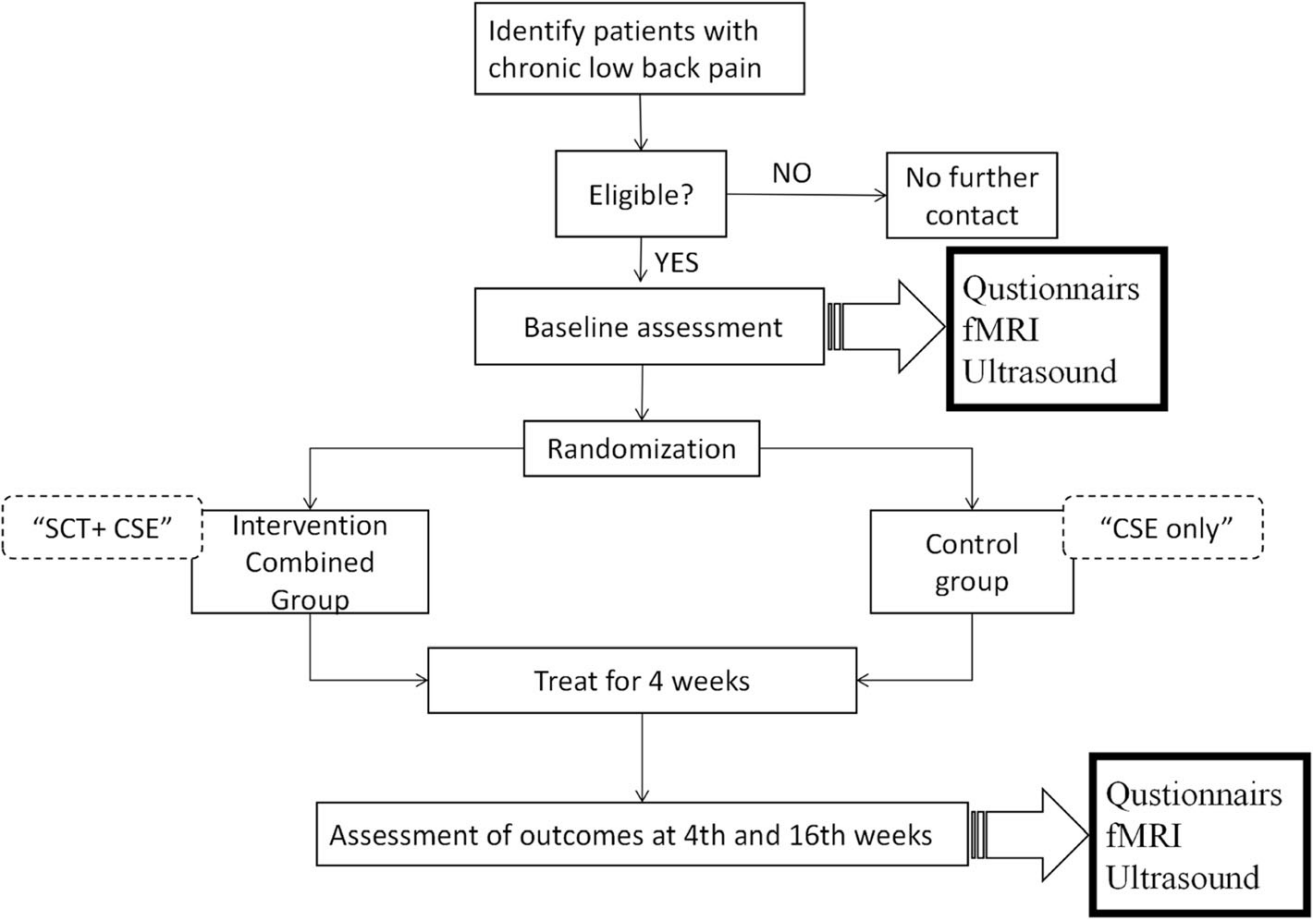
Flowchart of the trial protocol. SCT, self-compassion training; CSE, core stability exercise

The trial was designed as a randomized, controlled, observer- and patient-blinded trial with two parallel groups. Randomization will be performed as block randomization with a 1:1 allocation. The control group will receive CSE only while the intervention combined group will receive a combination of CSE and SCT. Participants will be asked to complete a follow-up assessment at weeks 4 (post-treatment) and 16 (primary end point) after randomization (Fig. 1).

The trial protocol will be conducted and reported according to the Standard Protocol Items: Recommendations for Interventional Trials (SPIRIT 2013) [20]. The study was prospectively registered with the Chinese Clinical Trial Registry, Number ChiCTR2100042810 (1/21/2021).

## Methods: participants, interventions, and outcomes

### Study setting {9}

The study site is located in the outpatient Department of Rehabilitation of the First Affiliated Hospital of Sun Yat-sen University, and participants in Guangzhou will be mainly recruited for the study.

The form of intervention will be group intervention with 10 participants per intervention combined group.

### Eligibility criteria {10}

Participants aged 18 to 60 years whose back pain has persisted for at least 12 weeks and are interested in participating in the trial will be recruited. Table 1 lists the exclusion criteria and the rationale for each criterion. The main screening criteria will be included in the registration questionnaire. We will contact possible eligible participants by telephone or WeChat and invited them to the hospital for final inclusion. In addition, if the participant does not receive treatment at the beginning or does not provide feedback data, we will define it as a drop out.

**Table 1:** Exclusion criteria

Exclusion criteria	Rationale
Low back pain has lasted < 3 months	Not NCLBP.
There were clear “red flag signs” (unilateral leg pain and numbness consistent with nerve distribution, musculoskeletal-originated pain, intermittent claudication, weight loss without an obvious cause, back pain at night, trauma, etc.)	Our interventions may not be effective.
The history of spinal surgery	Back problem is complicated by medical or medicolegal issues.
A score < 4 on Roland-Morris Low Back Pain and Disability Questionnaire (RMDQ)	Back pain too mild to detect improvement.
Patients who practice meditation regularly (weekly) in past 3 months	Possible bias due to current or recent interventions.
Patients who participated in courses related to core stability exercises in the past 3 months	Possible bias due to current or recent interventions.
Conditions that may be uncontrolled for self-compassion meditation (e.g., psychosis, major depression or anxiety, current self-harm, or suicidal ideation)	Condition would make it difficult to control or cause harm to participants.
Unable to independently complete Chinese language questionnaires	Condition would make it difficult to communicate.

### Who will take informed consent? {26a}

A study researcher (ZFM) will introduce the trial to potential participants and discuss the trial with them. If the patient agrees, the researcher will obtain a written consent form stating that the patients are willing to participate in the trial.

### Additional consent provisions for collection and use of participant data and biological specimens {26b}

Additional fMRI and musculoskeletal ultrasound will be needed to assess the changes in brain function and muscle structure and function.

### Interventions

#### Explanation for the choice of comparators {6b}

CSE is the traditional treatment for low back pain. However, many patients have poor adherence to exercise training without supervision. One of the aims of the study is to demonstrate that SCT can improve CSE adherence. Its selection as a comparator is therefore justified.

#### Intervention description {11a}

**Fig. 2:**
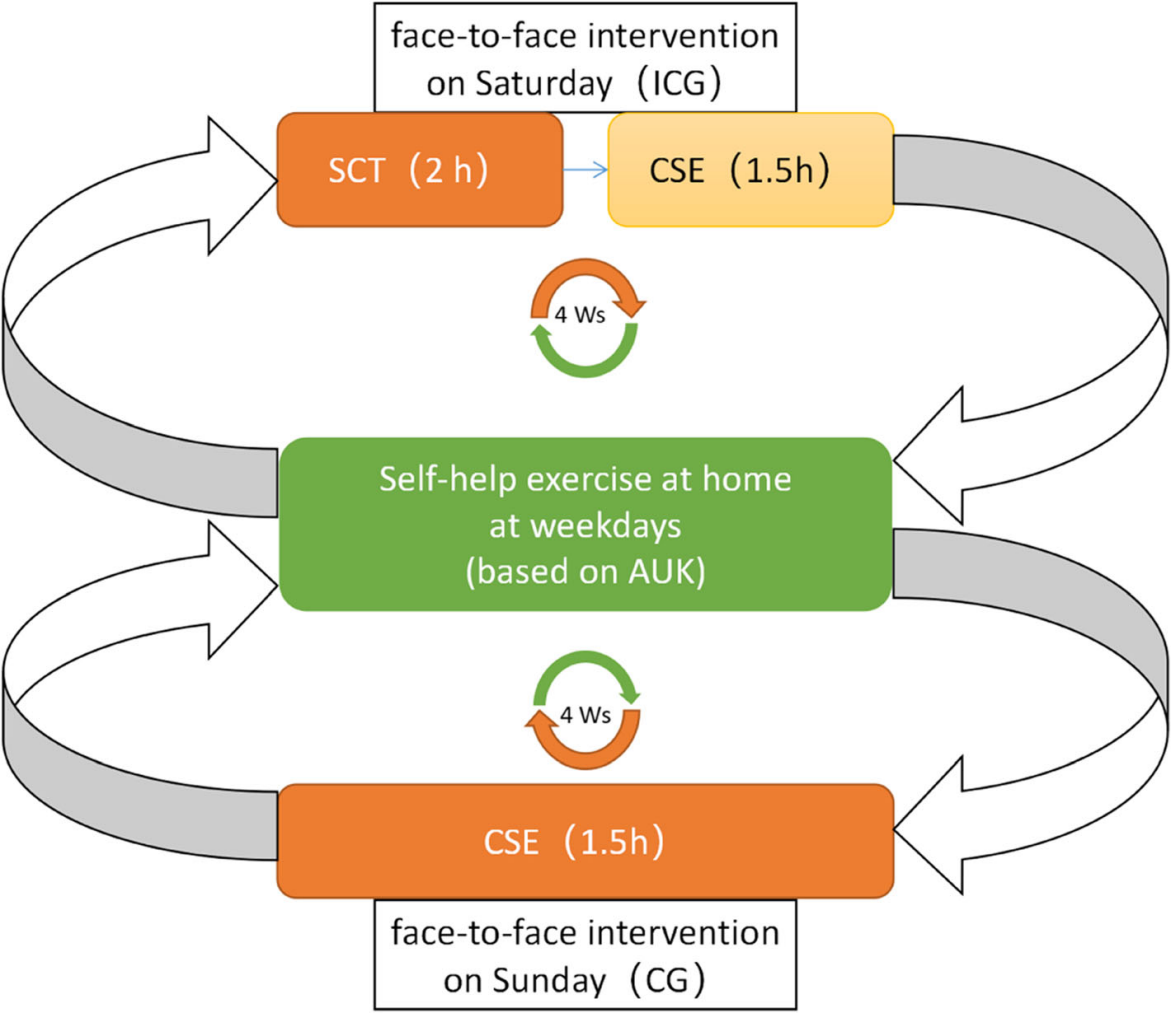
Flowchart of the intervention. SCT, self-compassion training; CSE, core stability exercise; ICG, intervention combined group; CG, control group

We will randomize participants with NCLBP into two groups: the intervention combined group (ICG) will receive m-health-based SCT plus CSE (the same day), and the control group (CG) will receive m-health-based CSE only. The treatment will be divided into two parts: face-to-face intervention and self-help exercise at home. The face-to-face intervention will take place in the Rehabilitation Clinic of the First Affiliated Hospital of Sun Yat-Sen University on Saturday or Sunday for 4 weeks in total, because most NCLBP patients need to work on the weekdays. The self-help exercise was provided by the Anyoukang (AUK) exercise training system, which would provide daily exercise reminders and video guidance via WeChat (Fig. 2).

#### Self-compassion training (SCT)

Two hours of SCT will begin in a group counseling room in the hospital. This intervention is adapted from our previous study [21] with some minor modifications based on the characteristics of NCLBP. During the intervention, group leaders will introduce information about self-compassion and guided participants through a series of practices. Participants will also be encouraged to do home practices following the guidelines provided by the investigators. Psycho-education about self-compassion will be provided in the first session, and a few practices to improve participants’ awareness of stress (e.g., observing body sensations under NCLBP) will be presented. The second session will seek to advance participants’ understanding of self-compassion using exercises that savored the experience accompanied by self-compassion practices (e.g., affectionate breathing meditation). The third session will focus on guiding participants to meet stress with self-compassion (e.g., loving-kindness meditation for ourselves). In the last session, participants will discuss and review their experience during the intervention with the group and make plans for future self-compassion practice (Table 2). SCT will be co-led by two graduate students in counseling psychology who have an adequate theoretical understanding of self-compassion and practiced mindfulness and self-compassion themselves. The whole intervention process will be supervised by a counseling psychologist (WYY).

**Table 2:** Content of core stability exercise and self-compassion training class sessions

Session	Core stability exercise		Self-compassion training	
	Theme	Content	Theme	Content
**1**	Stretching exercise	● Rolling spine exercise ● Lumbar rotation ● lumbar extension ● Cat stretch	Back to body sensations	● Self-introduction ● Body scan meditation ● Introduction about self-compassion ● Self-compassion touch
**2**	Add simple core strength exercise	● Hallowing training ● Hip bridge ● Simplified bird dog ● Half plank	Experience compassion	● The relationship between the three emotional systems (threat system, drive-reward system, soothing system and self-compassion) ● Affectionate breathing ● Compassionate image
**3**	Add intensive core strength exercise	● Inverted bicycle ride ● Hip-single leg support ● Bird dog ● Dead bug	Self-compassion under pressure	● Discussion about home practice ● Inner child ● Self-compassion writing
**4**	Summary	Develop personalized exercise programs	On the road	● Brief loving-kindness meditation ● Self-compassion phrases ● Review and Share ● Wishing bottle

#### Core stability exercise (CSE)

**Fig. 3:**
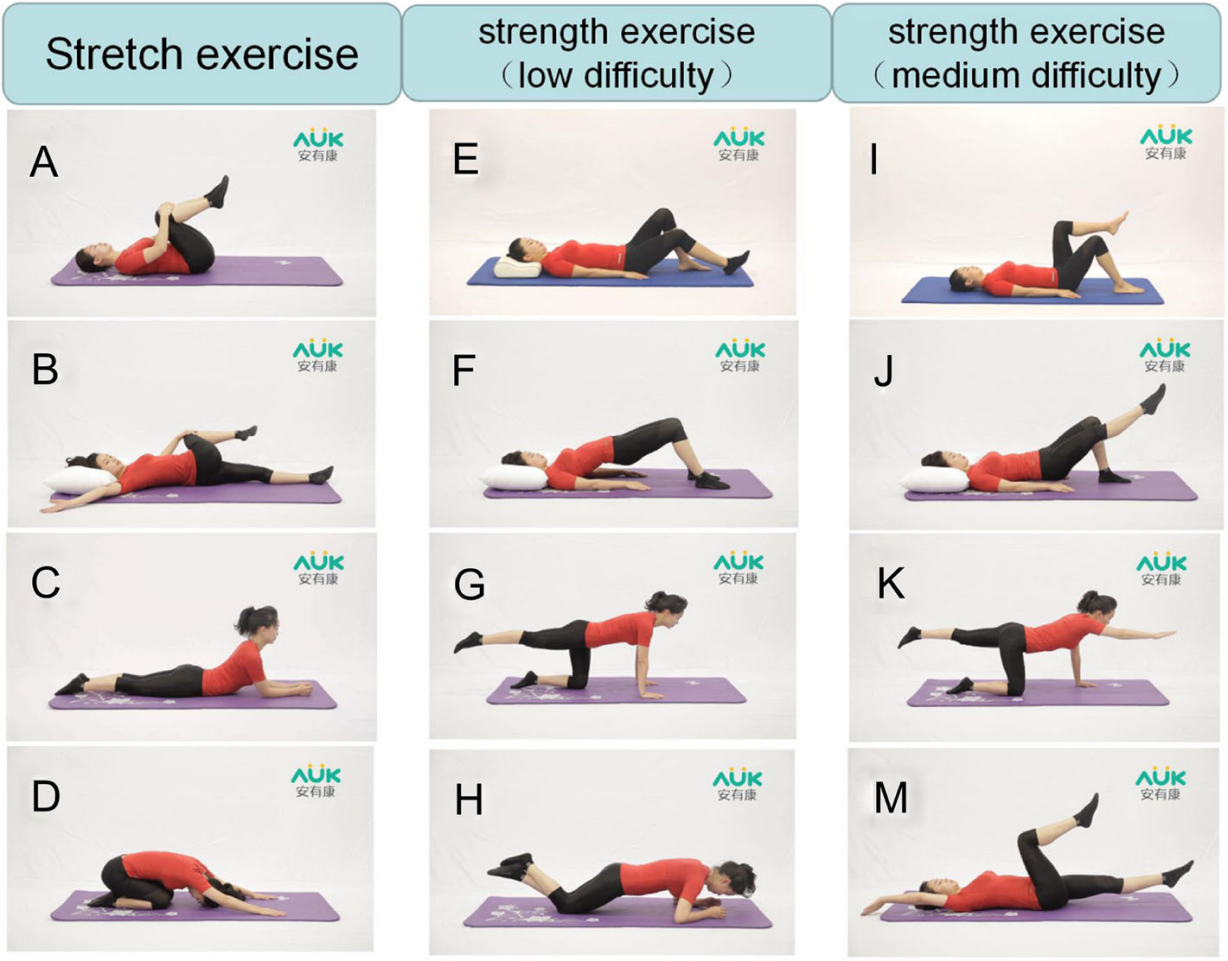
Exercises used in the SCE program. **A** Rolling spine exercise. **B** Lumbar rotation. **C** Lumbar extension. **D** Cat stretch. **E** Hallowing training. **F** Hip bridge. **G** Simplified bird dog. **H** Half plank. **I** Inverted bicycle ride. **J** Hip-single leg support. **K** Bird dog. **M** Dead bug

The face-to-face group core stabilization training of 1.5 h will be conducted under the supervision and guidance of professional physiotherapists on weekends. After the training, homework will be arranged for the subjects using the AUK Sports Training System. The participants will be asked to complete at least three home-based training sessions per week with a training time of 20 to 30 min [22] and to complete the daily training diary after training. The overall training lasted for 4 weeks. The first week will be spent mainly teaching the participants stretching exercises. In the second week, low-difficulty strength training will be added on top of the stretching training. In the third week, the participants will be taught more difficult strength training. In the last week, according to the actual condition of the participants, a set of exercise programs suitable for them will be determined (see Table 2 and Fig. 3). The program will be led by two professional physiotherapists with at least 5 years of experience (Liu Shufeng and Zheng Yiyi).

#### Criteria for discontinuing or modifying allocated interventions {11b}

Excessive exercise or inadequate warm-up will cause muscle pain (NRS > 7). In the event this happens, the participant should be told to reduce the intensity of the exercise or to rest for a few days.

#### Strategies to improve adherence to interventions {11c}

In addition to face-to-face therapy to improve adherence to the interventions, the participants will be asked to keep a diary after exercise, and they will be reminded every day through an electronic form as attached.

#### Relevant concomitant care permitted or prohibited during the trial {11d}

Relevant concomitant care and interventions (e.g., massage, physiotherapy modality, or medicine) are prohibited during the trial. This can affect the accuracy of treatment outcomes.

#### Provisions for post-trial care {30}

If participants are injured as a result of this study, they may receive free treatment and/or compensation in accordance with Chinese law in case of injury in connection with this clinical study.

### Outcomes {12}

Participants will complete the outcome assessment at baseline, the end of the 4th week, and the follow-up 16 weeks after baseline evaluation. Baseline data collection included demographic and clinical characteristics, such as gender, age, work status, height, weight, education, treatment history, and medical history. Participants scanned the QR code to complete the questionnaire by themselves.

#### Primary outcomes

The primary outcomes are disability associated with low back pain and pain intensity.

##### Back pain disability

Back pain disability will be assessed using the Roland-Morris Disability Questionnaire (RMDQ; scale 0–24; higher scores indicate greater functional limitation) [23].

##### Pain intensity

Pain intensity will be assessed with the Numeric Rating Scale (NRS), which measures the mean pain intensity during the last week (scale 0–10, higher scores indicate greater pain intensity) [24]. We selected three NRS types: average NRS (average pain intensity over the last week), current NRS (current pain intensity), and most severe NRS (most severe pain intensity over the last week) [16].

#### Secondary outcomes

The secondary outcomes will be measured using questionnaires, such as psychological status and general health status. The functional change in the lumbar core muscles after the intervention will be also evaluated.

##### Anxiety and depression symptoms

Anxiety will be measured with the 7-item Generalized Anxiety Disorder Scale (GAD-7; range, 0–21; higher scores indicate greater severity) [25]. Depressive symptoms will be assessed with the Patient Health Questionnaire-9 (PHQ-9; range, 0–27; higher scores indicate greater severity) [26].

##### Quality of life

Quality of life will be assessed with the 36-item Short-Form Health Survey (SF-36), which evaluates the health-related quality of life and is analyzed into 2 main domains, the physical component and the mental component [27].

##### Patient satisfaction

Patient satisfaction will be assessed using the Patient Satisfaction Questionnaire. This is a simple questionnaire asking patients how satisfied they are with their treatment with answers rated from 1 to 5 using the following criteria: 1 = satisfied, 2 = just a little satisfied, 3 = neither satisfied nor dissatisfied, 4 = just a little dissatisfied, and 5 = dissatisfied.

##### Brain function assessment

All imaging data will be acquired at the East Campus of Sun Yat-sen University using a 3.0-T MRI scanner (Siemens 3 T Prismas). Each participant will lay supine with their head snugly fixed with pillows and foam pads to reduce head motion. Participants will be asked to close their eyes and rest comfortably throughout the scans without moving or falling asleep. Functional MRI data will be acquired using a T2*-weighted, single-shot, gradient-recalled echo-planar imaging sequence with the following parameters: TR/TE = 2000/30 ms, field of view (FOV) = 224 mm × 224 mm, matrix size = 64 × 63, flip angle = 90°, 3.5 mm × 3.5 mm in-plane resolution, slice thickness = 3.5 mm, 33slices, and slice gap = 0.7 mm.

##### Lumbar core muscle function assessment

TrA and LM muscle thickness will be directly measured by ultrasound, and the thickness change rate between the contractile and resting states will be calculated to predict the postural stability from the perspective of muscle morphology [28]. Images of the LM and TrA will be acquired in B-mode with a portable ultrasound machine (KONICA MINOLTA Inc., SONIMAGE HS1, Japan). Images of the resting and contractile states of the TrA and LM at the L4 level will be taken by a physician familiar with musculoskeletal ultrasound.

For bilateral TrA muscles, maximum expiration will be performed while the participants maintained in a crook lying position. The muscle thickness will be measured while maximum expiration is maintained for 3–5 s. The TrA muscle will be measured three times, and the average value will be calculated [9]. Tree images on bilateral LM will be taken at rest to measure the thickness and cross-sectional area in a prone position. Next, participants will be asked to lift their limbs about 5 cm straight off the table for 3–5 s. Once a clear image of the LM is obtained, it will be frozen on the screen and saved [29].

The average resting and contractile thickness of the TrA and LM will be calculated using the ImageJ software (Media Cybernetics, Silver Spring, USA). The following calculation formula of muscle contraction rate will be used: contraction rate = (average contraction thickness − average resting thickness)/average resting thickness × 100%.

#### Potential mediators

The clinical effect of low back pain may be mediated by different variables, and the effects of all potential mediators on the outcomes will be explored in both treatment groups. The potential mediators of outcome will be assessed at baseline and at 4 and 16 weeks after randomization with brief screening questions for pain catastrophizing, as well as pain acceptance, self-compassion, and self-efficiency.

##### Pain catastrophizing

Pain catastrophizing will be assessed with the Pain Catastrophizing Scale (PCS). PCS consists of 13 items, and each item will be answered with a numeric value between 0 and 4; 0 corresponded to “not at all,” and 4 corresponded to “all the time.” Higher scores indicate a higher level of pain catastrophizing [30].

##### Pain acceptance

Pain acceptance will be measured with the Chronic Pain Acceptance Questionnaire-8 (CPAQ-8). The outcome represents the mental influence of pain. Two factors are contained in the scale: activity engagement and pain willingness. Higher scores indicate higher levels of pain acceptance [31].

##### Self-efficiency

Self-efficiency will be assessed with the Pain Self-Efficiency Questionnaire (PSEQ). The PSEQ consists of 10 items and assesses the extent to how confident the participant is in performing a range of certain activities using a 7-point Likert scale, with 0 denoting no confidence at all and 6 denoting complete confidence. A higher total score after adding up the score of each item indicates stronger confidence in mastering self-efficiency [32].

##### Self-compassion

Self-compassion will be measured with the Self-Compassion Scale (SCS). The scale is used to assess the degree that one realizes their suffering and wants to soothe oneself. The scale consists of 26 items measured on a 5-point scale with 1 = almost never and 5 = almost always; a higher score indicates a higher level of self-compassion [33].

### Intervention-related information

Intervention-related information will be evaluated by class attendance, adverse events, and exercise adherence. Class attendance will be assessed by class records. Exercise adherence and adverse events will be recorded using the home practice diary.

### Participant timeline {13}

The participant timeline is presented in Table 3.

**Table 3:** Content of baseline and follow-up questionnaires

Measures	Baseline	Week		
		1–4	4	16
**Baseline characteristics**				
**Patient characteristics** (age, gender, education, work status, BMI, pain duration)	X			
**Primary outcome**				
Back pain disability (RMDQ)	X		X	X
Pain intensity (NRS; average pain, worst pain, average pain)	X		X	X
**Secondary outcomes**				
Quality of life (SF-36)	X		X	X
Depression (PHQ-9)	X		X	X
Anxiessty (GAD-7)	X		X	X
Patient satisfaction			X	
Brain function assessment	X		X	X
Musculoskeletal assessment	X		X	X
**Potential mediators**				
Pain catastrophizing (PCS)	X		X	X
Pain acceptance (CAPQ-8)	X		X	X
Pain Self-Efficiency Questionnaire (PSEQ)	X		X	X
Self-compassion (SCS)	X		X	X
**Intervention-related information**				
Class attendance		X		
Exercise adherence		X	X	X
Adverse events		X	X	X

### Sample size {14}

Sample size calculation will be based on the results of our pilot study [34]. We found a mean difference of 5.5 for RMDQ with a standard deviation of 4.7 in the intervention group and a mean difference of 3.1 with a standard deviation of 4.8 in the control group at 16-week follow-up. To detect it at 16 weeks with a two-sided significance level (alpha) of 0.05 and a power of 80% with equal allocation to two arms, it would require 66 patients in each arm of the trial. To allow for 20% drop out, future study must include at least 166 participants (83 in each group). The calculation was performed with the PASS software(version 15).

### Recruitment {15}

Participants will be recruited in eight waves of 20 participants each. Recruitment will be advertised in notices posted in the clinic and on a social network platform (WeChat). The main inclusion and exclusion criteria will be included in our registration questionnaire. Possible eligible participants will be invited to come to the hospital for a physical examination and baseline assessment. Eligible participants will be assigned a number according to the sequence of their arrival at the hospital and a to the research assistant (YJJ) for random grouping. Participants will be informed of the time of treatment after baseline assessment, so that the participants could arrange the time. Treatment content will be announced 3 days before the start of the intervention.

### Assignment of interventions: allocation

#### Sequence generation {16a}

For the randomization, a simple block randomization process was conceived and will be implemented by a trial assistant (Yang Jiajia). After eligibility is confirmed, every participant will be assigned a unique number as an identifier. Sequence generation will be achieved using the IBM Statistical Package for Social Sciences (SPSS) version 23 software and stratified with a 1:1 allocation random block size of 10. The randomization list reports a progressive randomization number for randomized participants (from 1 to 20), and the treatment (SCE or combined SCE + CST) will be assigned to the subject in either group.

#### Concealment mechanism {16b}

For allocation concealment, the randomization list will be maintained by the trial assistant (YJJ) who would not participate in the entire treatment procedure.

#### Implementation {16c}

The trial assistant (YJJ) who was not involved in the entire treatment procedure generated and maintained the allocation sequence. The principal investigator (ZFM) enrolled and assigned the participants to the groups according to their unique numbers. Once allocated, the participant was not allowed to change their groups.

### Assignment of interventions: blinding

#### Who will be blinded {17a}

This method will provide 2-blinded conditions. During the intervention, participants will only know the participants in their own treatment group and will not know the identity of participants in the other groups. Each group will receive treatment at separate times to prevent contact between participants of the two groups. The outcome assessor (LY) and data analyst (LZC) will also be blinded to the group allocation. The physiotherapists and psychologists cannot be blinded to the group allocation due to the study design.

#### Procedure for unblinding if needed {17b}

Not applicable. There is no need for unblinding.

### Data collection and management

#### Plans for assessment and collection of outcomes {18a}

The data will be collected by the electronic questionnaire and automatically stored in the network disk database (https://mhlab7.wjx.cn/corplogin.aspx). The data will be jointly managed by the research team and analyzed by the statistical analyst, who will not know the grouping situation and intervention measures.

#### Plans to promote participant retention and complete follow-up {18b}

At the time of baseline assessment, participants will be charged a deposit of 100 yuan, which will be returned upon completion of the follow-up. The participants must complete two follow-up visits to receive their deposit (20 yuan for the first visit and 80 yuan for the second).

#### Data management {19}

Data will be collected from participants using the electronic questionnaire; they will fill in the form themselves according to the instructions. The data will be stored directly in the network disk of Wenjuanxing (https://mhlab7.wjx.cn/corplogin.aspx). After the patient fills it out, they are not allowed to modify it. The musculoskeletal ultrasound and fMRI data will be input into the computer by two people independently and then checked by a third one. If there is any inconsistency, it needs to be discussed and confirmed.

#### Confidentiality {27}

The personal information and confidentiality of the participants will be protected before, during, and after the trial. In all the saved data, only the unique number identifier of participants will be displayed, not the name of the participants. Only the researcher knows the name corresponding to the number.

#### Plans for collection, laboratory evaluation, and storage of biological specimens for genetic or molecular analysis in this trial/future use {33}

Not applicable. No biological samples will be collected for genetic or molecular analysis as part of this trial.

## Statistical methods

### Statistical methods for primary and secondary outcomes {20a}

We will use an intent-to-treat approach to analyze all available data at baseline, post-treatment, and 3 months. We will compare the baseline demographic and clinical characteristics of the groups using the independent *t*-test for continuous variables and chi-square tests for categorical variables. The primary endpoint is change in RMQD during 3 months. Analyses of the primary and secondary continuous outcome variables will be analyzed using two-way repeated-measures ANOVA. The effect sizes of the mean group differences will be calculated as the Cohen *d*. Differences with a 2-sided *P*-value < 0.05 will be considered significant. All data will be analyzed using IBM SPSS Statistics V.22.

### Interim analyses {21b}

An interim analysis will be performed on the primary endpoint after the first cohort (*n* = 20) of patients has been randomized and has completed the 3-month follow-up. The interim analysis will be performed by an independent statistician blinded to the treatment allocation and will report their findings to the project leaders (WCH and WYY). Statistical results will determine whether the trial should be continued, modified, or halted earlier.

### Methods for additional analyses (e.g., subgroup analyses) {20b}

Changes in the primary outcomes (RMDQ and NRS) will be correlated with other results (deep trunk muscle function, brain function, and potential mediators) to identify the reasons behind any change in the primary outcomes.

### Methods in analysis to handle protocol non-adherence and any statistical methods to handle missing data {20c}

We will use an intent-to-treat approach to analyze all available data; all participants will be assessed at baseline prior to randomization, so for the missing data, we will use the methods of the last observation carried forward or baseline observation carried forward to fill up.

### Plans to give access to the full protocol, participant-level data, and statistical code {31c}

According to the data sharing statement, we will deliver a completely deidentified data set to an appropriate data archive for sharing purposes no later than 3 years after the collection of the 1-year post-randomization interviews.

### Oversight and monitoring

#### Composition of the coordinating center and trial steering committee {5d}

ZFM, XWH, ZSY, WYY, and WCH comprise the steering committee.

#### Composition of the data monitoring committee, its role, and reporting structure {21a}

A Data Monitoring Committee (DMC) is not needed, because our trial is of short duration and with known minimal risks. But the researchers (ZFM and XWH) will regularly analyze the data to make adjustments.

#### Adverse event reporting and harms {22}

In this study, physical discomfort due to exercise will be defined as an adverse event. In the treatment process, the participants will be asked to fill in a training diary every day to report their physical conditions, so as to detect adverse events and deal with them in a timely manner.

#### Frequency and plans for auditing trial conduct {23}

We plan to conduct an audit every 4 weeks. The processes reviewed can relate to participant enrollment, consent, eligibility, and allocation to study groups and adherence to the trial interventions and policies to protect participants, including reporting of harms and completeness, accuracy, and timeliness of data collection.

#### Plans for communicating important protocol amendments to relevant parties (e.g., trial participants, ethical committees) {25}

Any modifications to the protocol which may impact the conduct of the study and potential benefit of the patient or may affect patient safety, including changes of study objectives, study design, patient population, sample sizes, study procedures, or significant administrative aspects will require a formal amendment to the protocol. We will first submit the change request to the ethics committee of the First Affiliated Hospital of Sun Yat-sen University and then implement the new plan after it is approved.

#### Dissemination plans {31a}

The study results will be released to the participants, healthcare professionals, the public, and other relevant groups via publication.

## Discussion

This trial attempts to combine SCT with CSE to improve the treatment of NCLBP. Since SCT not only relieves the negative emotions of patients with low back pain but also promotes healthy behaviors, we think it is therefore likely to improve the effect of CSE in treating NCLBP. A face-to-face intervention will only occur once a week; for the rest of the time, we will use social networks to communicate with the subjects. However, due to the influence of COVID-19, our face-to-face treatment may be suspended at any time, so we will consider developing an app for remote treatment in the future, making the treatment safer and more convenient for the participants.

## Trial status

Protocol version number: 5 (August 30, 2021)

First day of recruitment: April 15, 2021

Expected end of recruitment: October 30, 2023

## Abbreviations

NCLBP: Non-specific chronic low back pain
CSE: Core stability exercise
SCT: Self-compassion training
RMDQ: Roland-Morris Disability Questionnaire
NRS: Numeric Rating Scale
LM: Lumbar multifidus
TrA: Transversus abdominis
MBSR: Mindfulness-based stress reduction
GAD-7: 7-item Generalized Anxiety Disorder Scale
PHQ-9: Patient Health Questionnaire-9
SF-36: 36-item Short-Form Health Survey
PCS: Pain Catastrophizing Scale
CPAQ-8: Chronic Pain Acceptance Questionnaire-8
PSEQ: Pain Self-Efficiency Questionnaire
SCS: Self-Compassion Scale
